# Supplementary material for: Feasibility and acceptability of the Indian Autism Screening Questionnaire in clinical and community settings
Source: PLoS One. 2023 Nov 30;18(11):e0292544. doi: 10.1371/journal.pone.0292544 (PMC10688706; doi:10.1371/journal.pone.0292544)
Supplement: S1 Table — (DOCX) [file pone.0292544.s001.docx]

**Supplementary Table 1: Comparison of children with autism and without autism on sociodemographic and developmental factors in the Psychiatry OPD sample**

|  | No autism (n=34) | Autism(n=111) | F/χ^2^ value | p value |
| --- | --- | --- | --- | --- |
| Age | 11.71 (4.51) | 10.96 (5.20) | 0.562 | 0.455 |
| Gender Male/Female | 24 (71%)/  10 (29%) | 90 (81%)/  21 (19%) | 1.705 | 0.232 |
| Education | 3.44 (3.81) | 2.78 (3.92) | 0.740 | 0.391 |
| Currently studying Yes/No | 4 (95%) /  30 (5%) | 23 (21%) /  88 (79%) | 1.378 | 0.318 |
| Father’s current age | 40.35 (6.87) | 43.19 (6.66) | 3.15 | 0.079 (NS) |
| Mother’s current age | 36.42 (7.19) | 39.64 (6.38) | 4.33 | 0.040 |
| Father’s age at birth of child | 29.09(6.18) | 31.80(6.69) | 2.98 | 0.088 |
| Mother’s age at birth of child | 24.79(6.25) | 28.26(6.37) | 5.39 | 0.022 |
| **Father’s years of education** | **10.76 (4.79)** | **13.79 (4.24)** | **12.48** | **0.001** |
| **Mother’s years of education** | **10.56 (5.20)** | **13.46 (4.70)** | **9.440** | **0.003** |
| Consanguinity  Absent/Present | 33 (97%)/  1 (3%) | 109(99%)/  1(1%) | 0.783 | 0.418 |
| Family History Absent/Present | 25(74%)/  9(26%) | 84(77%)/  261(23%) | 1.11 | 0.82(NS) |
| Type of Pregnancy  Uneventful/Eventful | 28(90%)/  3(10%) | 93(84%)/  18(16%) | 0.822 | 0.414(NS) |
| Nature of delivery  Normal/Caesarean | 27(84%)/  5(16%) | 64(58%)/  47(42%) | 7.662 | 0.006 |
| Pre-natal Complications  Absent/Present | 29(95%)/  3(5%) | 90/81%)/  21(19%) | 1.62 | 0.285 |
| Natal Complications  Absent/Present | 27(84%)/  5(16%) | 93(84%)/  18(16%) | 0.006 | 1.00 |
| Post-natal Complications  Absent/Present | 18(56%)/  14(44%) | 64(58%)/  46(42%) | 0.038 | 1.00 |
