# Supplementary material for: Feasibility and acceptability of the Indian Autism Screening Questionnaire in clinical and community settings
Source: PLoS One. 2023 Nov 30;18(11):e0292544. doi: 10.1371/journal.pone.0292544 (PMC10688706; doi:10.1371/journal.pone.0292544)
Supplement: S2 Table — (DOCX) [file pone.0292544.s002.docx]

**Supplementary Table 2: Comparison of children with autism and without autism on sociodemographic and developmental factors NIEPD sample***

|  | No autism(n=64) | Autism(n=110) | F/χ^2^ value | p value |
| --- | --- | --- | --- | --- |
| Age of the child | 12.28(3.33) | 8.70 (3.51) | 43.71 | <0.0001 |
| Gender Male/Female | 28(44%)/  36(56%) | 76 (69%)/  34 (31%) | 10.80 | 0.001 |
| Education of the child | 5.69 (3.45) | 1.24 (2.68) | 89.79 | <0.0001 |
| Child currently studying Yes/No | 1(2%)/  62 (98%) | 17 (15%)/  93 (85%) | 8.26 | 0.003 |
| Father’s current age | 42.02(5.58) | 39.37(6.00) | 7.48 | 0.007 |
| Mother’s current age | 37.07 (5.12) | 36.81(9.85) | 0.035 | 0.85 |
| Father’s years of education | 7.17(5.71) | 13.00(4.99) | 49.63 | <0.0001 |
| Mother’s years of education | 6.61(5.95) | 12.86(5.05) | 54.37 | <0.0001 |
| Father’s age at birth of child | 30.05(5.58) | 30.67(5.18) | 0.49 | 0.48 |
| Mother’s age at birth of child | 24.85(5.32) | 28.12(9.68) | 5.92 | 0.016 |
| Consanguinity  Absent/Present | 57(93%)/  4(7%) | 104(96%)/  4(4%) | 0.71 | 0.46 |
| Family History Absent/Present | 55(90%)/  6(10%) | 89(82%)/  19(18%) | 1.86 | 0.19(NS) |
| Type of Pregnancy  Uneventful/Eventful | 43(72%)/  17(28%) | 103(95%)/  5(5%) | 19.04 | <0.0001 |
| Nature of delivery  Normal/Caesarean | 49(82%)/  11(18%) | 66(61%)/  43(39%) | 7.94 | 0.006 |
| Pre-natal Complications  Absent/Present | 56(93%)/  4(7%) | 91(83%)/  18(17%) | 3.31 | 0.094 |
| Natal Complications  Absent/Present | 53(88%)/  7(12%) | 92(86%)/  15(14%) | 0.186 | 0.813 |
| Post-natal Complications  Absent/Present | 41(56%)/  19(44%) | 60(58%)/  48(42%) | 2.63 | 0.105 |

*in DAV school, there was only one child with autism, who has not been included here.
