# Supplementary material for: Feasibility and acceptability of the Indian Autism Screening Questionnaire in clinical and community settings
Source: PLoS One. 2023 Nov 30;18(11):e0292544. doi: 10.1371/journal.pone.0292544 (PMC10688706; doi:10.1371/journal.pone.0292544)
Supplement: Attachment — Submitted filename: Comments.docx [file pone.0292544.s005.docx]

**Comments**

**Introduction**

- There is mention of Indian surveys in autism, it would add value if you can bring in figures from the Indian surveys on prevalence and incidence rate of autism in India.
- Would be great for a reader to understand the broad ten components of IASQ.
- Line 107- It is used by primary health worker…..? in which country is it used? In which sector it is used by primary health workers? Are there any government programs or schemes which helps in early detection and identification of Autism in India. If any, please mention the tool administered by them?
- To increase the readability, it would be great to mention the five diverse samples briefly based on the setting you are going to test the tool.
- Please brief on the research question: Importance of testing the tool beyond psychiatric setting as the authors have mentioned it as free, simple and easy to train tool in line 104

**Methods**

- Ethics permissions- please mention the name of IEC with date and reference number, may be crucial as children are involved. Also mention about the additional permissions required for the study
- How was the consent from psychiatric setting obtained

**Training**

- Was the training in person or online, and how many days and how many participants, period of training? Qualification of researches trained?

**Setting**

- Mention the period of recruitment and data collection.
- Please mention how was the data collected data managed and privacy was ensured

**Results**

Table 1- community primary school n=41 please check n. if n=40 then overall N has to be reduced to 916

**Discussion**

- Please bring in context of LMICs to the discussion part
